# Analysis of Non-Metallic Inclusions by Means of Chemical and Electrolytic Extraction—A Review

**DOI:** 10.3390/ma15093367

**Published:** 2022-05-07

**Authors:** Shashank Ramesh Babu, Susanne Katharina Michelic

**Affiliations:** Christian Doppler Laboratory for Inclusion Metallurgy in Advanced Steelmaking, Montanuniversität Leoben, 8700 Leoben, Austria; susanne.michelic@unileoben.ac.at

**Keywords:** chemical extraction, electrolytic extraction, non-metallic inclusions, secondary metallurgy

## Abstract

Research on non-metallic inclusions is of critical importance, as they have a significant influence on the final properties of steel products. In this regard, the 3D analysis of inclusions isolated from steel samples allows for the accurate measurement of their chemical composition, without the influence of the steel matrix, and detailed insights into their morphology. Inclusions can be extracted from the steel sample matrix using extraction methods followed by their measurement with scanning electron microscopy. Extraction methods can be broadly classified into chemical and electrolytic analyses. There have been numerous studies documenting the different extraction methods for the isolation of different inclusion types in a range of steels. The focus of this paper is to briefly review their developments over a century up until the present period. The most relevant methods and the corresponding steels and observed inclusions are also summarized in a table which could be a useful reference for researchers in this field.

## 1. Introduction

Non-metallic inclusions in steels have been of deep interest to researchers for over a century, as they have been found to impact the final properties of steel products. The type, size and distribution of non-metallic inclusions present in the steel matrix are intimately connected to behaviour of steel, especially when it is used in demanding applications [1]. Inclusions can form and can be modified further along every step of the steelmaking process. Therefore, steel cleanness requires considerations regarding the selection of input material and physical and chemical reactions in systems consisting of steel, slag and refractories [2]. The term “steel cleanness” is used to describe the number, size, chemical composition, morphology and distribution of non-metallic inclusions in the steel matrix [2]. Steel cleanness is a vital issue for steel makers, suppliers and their customers, especially when there is a demand for a high steel quality [2]. Earlier work in non-metallic inclusions concerned efforts to reduce their content in steels during processing. Since the 1980s and Kay’s work on inclusion engineering [3], research on oxide metallurgy by Takamura and Mizoguchi [4] and work on inclusion engineering by Wijk [5], research has moved on towards the minimization of the deleterious effects of inclusions on steel properties and processing and the utilization of inclusions for optimizing the steel microstructure [2]. As this field offers significant opportunities and challenges towards the production of high-quality steels, the development of tools to reliably and effectively characterize inclusions are critical for answering questions in the field of inclusion engineering.

There have been several methodologies to characterize inclusions [6,7,8,9,10], which can be broadly classified as indirect and direct. Indirect methods involve measurements such as total oxygen analysis and the measurement of magnetic properties of the steel. Although these methods might leave room for interpretation, indirect methods can be a good indicator of changes in steel cleanness during processing [11]. Direct methods involve the determination of the size, number, shape, position and composition of inclusions [11]. The most commonly used tool in direct methods is the scanning electron microscope integrated with an energy dispersive spectroscopy detector (SEM-EDS). Extensive information, such as data on the morphology and composition of inclusions, can be gathered, which enables the comprehensive analysis of inclusions found in the steel. This analysis can be done manually by observing a region of interest on the cross section of a steel sample or by using the automated 2D method. The automated method can provide detailed and wide-range information of a large quantity of inclusions across the cross-section of a steel sample. This is considered a state-of-the-art tool for inclusion analysis [11,12,13,14,15,16]. The disadvantages of this method are that the reliable analysis of size measurements, size distributions and morphology are not possible when using 2D manual and automated methods. Moreover, the steel matrix influences the analysis of the composition of the inclusions, thereby providing an inaccurate measurement.

Therefore, the use of extraction techniques which dissolve the steel matrix, leaving behind the inclusions, is very important. Extracted inclusions allow for a 3D analysis which mitigates the several disadvantages of the 2D method. Extraction techniques can generally be divided into electrolytic and chemical methods. The electrolytic method involves the dissolution of steel in a galvanic cell using suitable electrolytes which are controlled at defined electric parameters. The steel sample functions as an anode and a relatively inert metal such as platinum or stainless steel acts as cathode. These are then immersed in a suitable electrolyte and subject to electrolysis. The steel sample anode dissolves into the electrolyte, leaving behind a residue. The non-metallic inclusions remain undissolved due to their difference of potential with the steel matrix [17]. Chemical extraction involves the dissolution of the steel matrix in either acids or halogen solutions, leaving behind the undissolved inclusions. The residues from both methods are filtered through micro-sieves so as to isolate the inclusions. Although both of these methodologies are very old, a topic which will be discussed in the next section, such extraction methods have recently gained attention, especially for their accurate characterisation of inclusion morphology. This is important for the evaluation of the origin of the inclusion and its evolution across the steel processing route, as described in the works of Zhang et al. [18] and Janis et al. [19].

The purpose of this work is to briefly review the different electrolytic and chemical extraction methods used over the years in a roughly chronological manner, to describe the important developments and tabulate the important methods for a various range of steels.

## 2. Chemical Extraction Methods

### 2.1. Early Developments (1900s–2000s)

The chemical extraction methods used to isolate inclusions which are mentioned in the work of Goto and Wantanabe [20] are the hot nitric acid and chlorine methods. In the hot nitric acid method, the samples are dissolved in nitric acid (1:3) and heated at 90 °C, which is similar to the set-up used by Kobayashi et al. [21]. The chlorine method involves the use of an improved apparatus based on the work of Woesmuth [22], Colbeck et al. [23] and Moriwaki [24], through the use of which the metallic elements react with chlorine and form as chlorides, leaving behind oxides. It was found that both the hot nitric acid and chlorination methods produce reproducible results for plain carbon steel (0.25 wt.% C). The nitric acid method produced scattered results for silicon steel, whereas the chlorine method produced reliable results for the inclusions in the steel. The chlorine method was also shown to be most time efficient of the methodologies detailed by Goto and Wantanabe owing to the fact that it took two hours to dissolute the steel. The hot nitric acid method took about seven days to dissolve the steel sample. Yamada [25] analysed iron oxide in sponge iron (0.65 wt.% C) by comparing the dissolution of metallic iron with respect to the aqueous iodine and mercuric chloride solution methods. It was found that the inclusion content was comparable between the two methods, but the aqueous iodine method allowed for the precise determination of iron oxides in the steel.

Walz and Bloom [26] documented the procedures for extracting non-metallic inclusions from steel via the process of dissolving the metallic components, leaving behind a non-metallic inclusion residue. It was stated that prior research on acid extraction methods [27,28,29,30] revealed that only Al_2_O_3_ and SiO_2_ could be successfully extracted, even though there was an appreciable loss of these constituents, and that acid extraction methods were not suitable for the complete and unaltered separation of the inclusions. Prior research on halogen methods [23,31,32,33,34,35,36,37,38] was also discussed. These methods involve liquid solution procedures using bromine and iodine and gaseous treatment such as the chlorination process, whereby only metallic constituents are converted into volatile chlorides under exposure to gaseous chlorine. Liquid halogen methods have been shown to be successful for the extraction of inclusions from unalloyed, low and medium carbon steels. The chlorine method was successfully implemented for plain-carbon and low alloy steels. The drawback of the chlorine halogen method is that cannot not be used for alloyed steels. This is because non-volatile chlorides such as chromium chlorides are formed at the elevated operational temperatures where chlorine starts to attack the oxides. 

Smerko and Flinchbaugh [39] reviewed chemical extraction techniques for the purposes of inclusion extraction. It was noted in the paper that, up to that point, chemical extraction techniques had been mostly used to extract oxides from steels. Acids such as HCl, H_2_SO_4_ and HNO_3_ [38] were found to be partially successful in extracting oxides such as alumina and silica. But a comprehensive analysis of most oxides was not possible due to them partially or completely dissolving in the above-mentioned acids. Inclusions such as (Fe,Mn)O and carbides were shown to be isolated from rimmed steels using nitric acid maintained at 5 °C with the help of dry ice [40,41]. This came with disadvantages such as surface oxidations and the loss of large inclusions when steel millings were used. Mori [42] showed that HCl at room temperature can be used to extract niobium carbides, niobium nitrides and niobium carbo-nitrides from high purity Fe-Nb-C, Fe-Nb-N and Fe-Nb-C-N alloys. Smerko and Flinchbaugh [39] discussed the displacement techniques in which an aqueous solution of a salt of a metal is placed in contact with another metal just above it [43]. The solution was selected based on the electromotive series so that sample metal replaces the metal ion of the salt solution [43]. The displacement method was used from the 1930s to the 1950s. It came with limitations such as the destruction of all inclusions except for stable oxides and the retardation of the reactions due to the formation of a coating on the unreacted part of the steel samples. Halogenation techniques, wherein gaseous halogens such as chlorine react with iron in steel, were also discussed. Innovations in these techniques were made by the Walther Koch group in Germany after 1935 and chlorination furnaces were regularly used for isolating inclusions at the August Thyssen Works in the early 1960s [39]. Although Koch et al. [44,45] had demonstrated the application of chlorination for the isolation of various inclusions, the method came with serious drawbacks, such as the inaccurate analysis of inclusions in steels with more than 0.5 wt.% C, the high possibility of the destruction of oxides and silicates, and the safety precautions required due to the handling of chlorine gas in a laboratory. Therefore, halogenation techniques were no longer as widely used by the late 1960s. Aqueous solutions of halogens such as iodine were among the first extraction methods to isolate inclusions, with their use dating back to 1868, as documented by Lundell et al. [46]. But this method was abandoned due to drawbacks such as the extraction of only stable oxides and errors during the analysis of iron and silicon. Aqueous solutions of bromine were also used to extract carbides from alloy steels [47]. This came with drawbacks such as the low solubility in water limiting the quantity of reagent for the dissolution of the iron matrix, increased acidity in water causing the dissolution of less-stable oxides and the contamination of isolated residues by the products of hydrolysis reactions [43]. When compared to the above-mentioned halogen-based methods, it was found that halogen-organic solvent systems were found to be useful for the extraction of the entire range of inclusion types and micro constituents in steels. Solvents such as iodine-methanol have been used since the 1930s, with the implementation of continuous improvements in techniques and applications up until the 1960s [31,48,49,50,51]. Iodine-methanol came with challenges such as slow reactions, the partial dissolution of less stable iron and manganese oxides due to treatment temperatures of 60–65 °C and difficulties in the extraction of specific oxides and other phases of interest. Smerko and Flinchbaugh [39] showed that ultrasonic agitation of the iodine-methanol solvent led to a rapid steel dissolution and that the experiments could be performed at lower temperatures than the previously mentioned experiments. Inclusions such as (Fe,Mn)O, Fe_3_C and MnS could be extracted from solid rimmed samples. The use of bromine-methanol developed based on the work of Garside and Rooney [31] on iodine-methanol, and the procedure was dominant during the 1960s. Okura [52] undertook extensive studies by comparing bromine-methanol and iodine-methanol solutions. It was found that bromine methanol was very good for extracting inclusions from killed steels because oxygen and water dissolved in methanol caused fewer problems, specific oxides could be extracted and better oxide recoveries could be made. However, the bromine methanol attacked the MnO inclusion found in rimming steels. Bohenstedt [53] further developed apparatus to handle the exothermic dissolution reactions when bromine-methanol was used, and an arrangement was made to add methanol dropwise. Beeghly [54] developed another method to extract AlN and other nitrides from simple steel compositions using bromine-methyl acetate. This was later extended to extract oxides from steels by Raybeck and Pasztor [55]. This solvent could be used to extract (Fe,Mn)O from both rimming and semi-killed steels in addition to all the phases which could be isolated using the bromine-methanol solvent. The apparatus and procedures are well described in the work of Smerko and Flinchbaugh [39].

Narita et al. [56] decomposed steel specimens in HCl, H_2_SO_4_ and H_3_PO_4_ at room temperature and H_3_PO_4_ in a water bath kept at 95° C. After the complete dissolution of the matrix, the residue was filtered out. It was found that TiC could be isolated by HCl and H_3_PO_4_, VC by HCl, and (Cr,Fe)_23_C_6_ and (Cr,Fe)_7_C_3_ by H_3_PO_4_ and Mo_2_C in H_3_PO_4_ at 95 °C.

Narita [57] detailed the different methods of extracting inclusions in the steel sample and detailed the chemical reagents used in both acid and halogen-alcohol methods. In particular, a description of the Dickenson method [27] was included, wherein the steel was decomposed in a 10% nitric acid stirred by air bubbling at room temperature. Dickenson successfully isolated and determined the silica and the silicate content after oxidizing and decomposing the carbides with potassium permanganate in the residue. But the drawback was that the Dickenson method takes about 2 to 6 weeks to complete the process [30]. To overcome the disadvantage of a long duration at room temperature, an improvement was suggested involving the introduction of a heating decomposition method [58]. This was widely applied to rapidly isolate silica and alumina in steel. Acids such as H_2_SO_4_ and HCl acid could also be used in the Dickenson method. Cunningham et al. [38] also described a modified Dickenson method wherein the strength of the nitric acid is increased (up to 20% nitric acid) and the liquid is not aerated. This method reduces the time required for the dissolution of steel so that it is much less than when using ferrous iodide or 20% hydrochloric acid [59]. Al_2_O_3_ can be extracted from steels containing manganese-aluminium-silicate using this method. It was reported that HNO_3_ was best suited for low carbon and low alloyed steels, but H_2_SO_4_ was found to be more suitable for high carbon and high alloyed steels. 

### 2.2. Current Progress (2000–Present)

**Figure 1:**
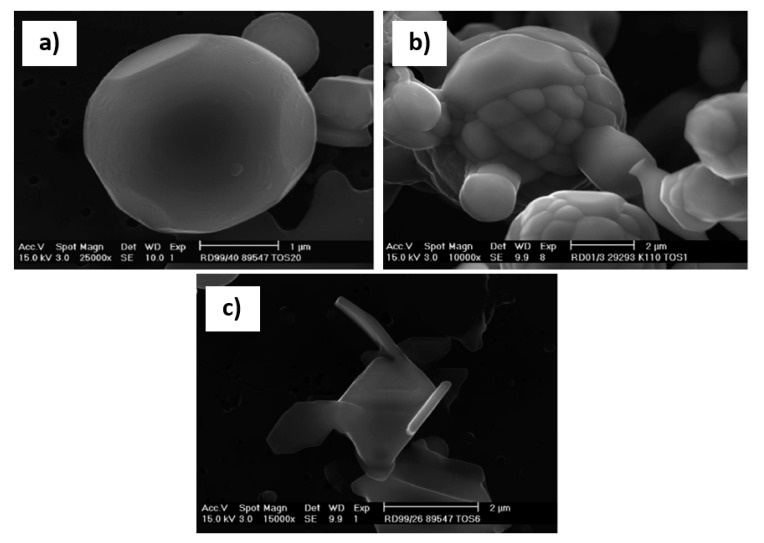
Different morphologies ((**a**) spherical, (**b**) agglomerated and (**c**) plate-like overgrowth) of alumina as extracted from a low carbon Al killed steel as mentioned in Dekkers et al. [60]. Copyright 2003, Springer Nature.

More recently, Dekkers [60] demonstrated the application of hot HCl in the process of extracting inclusions from low and medium carbon aluminium killed steels (C: 0 to 450 ppm, 250 to 450 ppm, 1100 to 1400 ppm). Dekkers used it to extract different morphologies of alumina from the samples seen in Figure 1. Fernandes et al. [61] demonstrated the use of HCl in the dissolution of the ferritic matrix of a continuously cast SAE 1015- Al deoxidised steel. This methodology was shown to work well for inclusions formed by- Al,Si, Ca, Mn and Mg, but sulphur containing inclusions were shown to completely dissolve by this method.

Lu et al. [62] also used the HCl electrolyte matrix (1:1 mixture HCl acid by volume and distilled water at temperatures of 338 to 343 K) to dissolve the ferrite matrix of Grade 100 micro-alloyed steel. Although chemical extraction was found to be more efficient in terms of the residue collected when compared to electrolytic extraction, a large quantity of SiO2 was found present in the chemically extracted residue.

Janis et al. [63] compared chemical and electrolytic extraction techniques for a 18/8 stainless steel (Fe-18 wt.% Cr-8 wt.% Ni) and a Fe-10 wt.% Ni alloy. It was found that halogen-alcohol solutions such as 5% bromine-methanol and 14% iodine-methanol had higher dissolution rates when compared to electrolytic methods. But it was not possible to obtain a stable dissolution rate, which is preferred for the reliable prediction of the aimed dissolved weight of the sample. The halogen-alcohol solutions were also about 5–10 times faster in terms of the layer dissolution of the sample when compared to electrolytic extraction techniques. Therefore, it was recommended to use such methods only in cases of short time dissolutions for situations such as sample etching. It was also mentioned that while stable oxides such as alumina can be extracted by chemical methods, unstable oxides such as CaO and MgO tend to dissolve in chemical acids and halogens [64,65,66]. 

**Figure 2:**
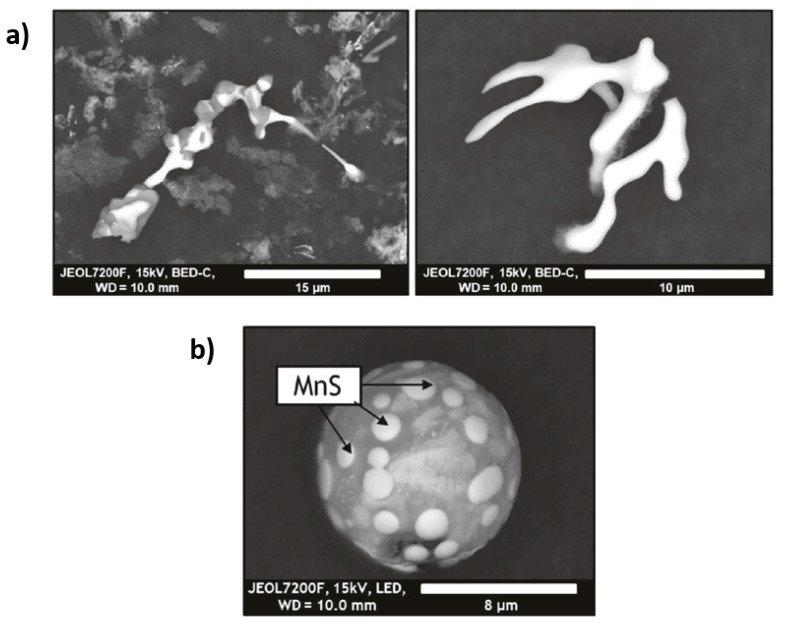
SEM images of (**a**) irregular shaped sulphide inclusions extracted from a plain carbon steel and (**b**) oxide inclusions with sulphide regions as extracted from low carbon high strength grade. The sulphides were extracted using the sequential chemical extraction using the 5% Nital. Adapted from the Ph.D thesis of Alexander Mayerhofer [11].

**Figure 3:**
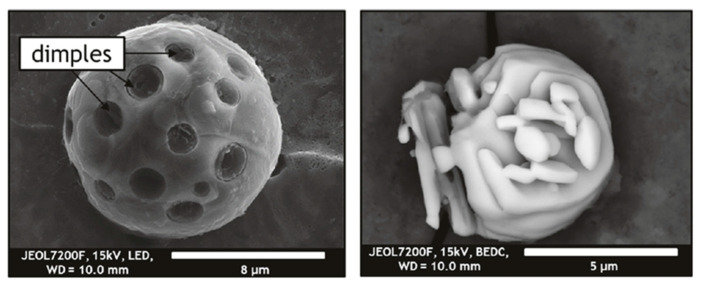
Oxide inclusions with missing sulphide regions which were dissolved after electrolytic extraction using the choline chloride solution. Adapted from the Ph.D thesis of Alexander Mayerhofer [11].

Recent work at Montanuniversitaet Leoben [11,67] demonstrated the use of sequential extraction with 5% Nital solution for the extraction of inclusions from steels, especially sulphides. The procedure of the sequential extraction is as follows: the steel sample is placed in a beaker with unagitated 5% Nital. After every 15 min, any visible residue around the sample is pipetted out and then injected into another beaker containing ethanol to arrest any further reaction. This is carried out for about 1.5 h. The contents collected using the pipette can also be filtered immediately to avoid any further reactions. This method has been found to be very successful in the extraction of a whole range of inclusion types and especially for the extraction of sulphides, as seen in Figure 2. The sequential extraction technique procedure is relatively simple, and the Nital solution is readily available in any metallurgical laboratory. In particular, sulphides can be extracted stably, especially from oxide-sulphide inclusions, when compared to electrolytic extractions performed when using choline chloride [68], as seen in Figure 3. Large agglomerates of sulphides can also be extracted using the sequential method. Some of the issues faced by this method is the detection of Si in EDS analysis, even in steels samples which are not alloyed with it, which was theorized as to have formed due to the acid reaction with the glass beaker. Another issue which has been reported is copper enrichment in the sulphides. This was theorized to be because of the very high affinity of copper ions for sulphur when the steel matrix in the sample is dissolved. The copper deposited could be clearly observed when the inclusions were observed under a SEM-EDS at low acceleration voltages. But this was not as pronounced when observations were made with relatively higher acceleration voltages. This suggests that the copper from matrix after steel dissolution appears to get deposited only as a thin layer on the surface of the sulphides. Steel specimens analysed using this methodology ranged from very low carbon to alloyed steels (0.002 to 0.21 wt.% C) [69].

## 3. Electrolytic Extraction Methods

### 3.1. Early Developments (1900s–1980s)

Some of the earliest attempts to extract inclusions using electrolysis methods were performed by the Koch group [70,71]. The experimental apparatus employed in their work was particularly complicated. The electrolyte used by the Koch group was a solution of 5% Sodium citrate, 1.2% Calcium bromide and 0.6% Calcium iodide in water. This was maintained at a pH of 8 by the addition of sodium hydroxide. It was shown that this could be used for steels whose alloy composition ranges from 0.01 to 1.2 wt.% C, 0.03 to 1 wt.% Mn and 0.01 to 1 wt.% Si. It was also observed that higher currents employed during electrolysis lead to the generation of hydrogen gas, which was problematic. Koch et al. [72] detailed potentiostatic methods for conducting the electrolysis of steel. In addition, Koch and Sundermann [73] showed how current density and potential curves depended on the composition of the steel given a particular electrolytic solution. Considering that the earlier works were focussed on the extraction of oxides, Artner [74] isolated sulphides such as titanium sulphide and zirconium sulphide from low carbon steels ranging from 0.025 to 0.1%. C. Born [75] successfully extracted sulphides from twenty steel specimens (ranging from 0.11 to 0.17 wt% C). The isolation methodologies employed in all the works mentioned above used the Koch group experimental apparatus using 5% sodium citrate electrolyte. Koch et al. [76] looked into the usage of sodium peroxide-based electrolyte for the extraction of inclusions such as nitrides and carbides using only a limited amount of raw material. Kroll and Lenk [77] developed a more economical, simpler and less fault-prone process for the purpose of the electrolytic extraction of oxides from steels when compared to the older apparatus developed by the Koch group. 

Goto and Wantanabe [20] investigated non-metallic inclusions in plain carbon (0.25 wt.% C) and silicon steels (0.08 wt.% C, 4.06 wt.% Si). They compared the use of both electrolytic extraction and chemical methods to isolate the inclusions. An electrolytic solution of ferrous sulphate was used for the dissolution experiments, which were conducted for about 24 h. The residue was then further treated with ferrous iodide-iodine solution to decompose the carbides. It was found that the electrolytic extraction gave scattered results in terms of inclusion contents for both the plain carbon and silicon steel samples when compared to chemical methods. The results of the chemical methods are detailed in the next section. 

Yoshida and Funahashi [78] reported the use of a simple ultrasonic sieving method and compared it with the complex elutriation method, which used to feature regularly in the years prior to their work in the isolation of inclusions from slime residues obtained from electrolysis experiments. They used a 10% ferrous chloride solution at a current of 5 to 10 A. This method took about 25 days to dissolve 4 kg of steel. The pH of the electrolyte was maintained between 3 and 5 by addition of HCl. Si and Al killed steels (0.04–0.2 wt.% C) were used as samples in this work. It was found that both methods were effective in the removal of inclusions of sizes less than 50 µm. The elutriation method was effective in the extraction of large inclusions from both Al and Si killed steels, whereas the ultrasonic sieving method was found to be effective only for Si killed steels. This was because the larger fragile alumina type inclusions which are found in Al killed steels tended to disintegrate under the ultrasonic sieving method.

Narita et al. [56] explored the potentiostatic electrolysis approach with the objective of isolating carbides from steel samples. The specimens used for the study were Fe-C binary steel and Fe-C-M ternary alloys (M: Ti, Zr, V, Nb, Cr, Mo or W). The C alloying composition was 0.05–0.4 wt.%. It was found that carbides such as Fe_3_C, VC, Mo_2_C and (Fe,W)_23_C_6_ can be isolated by using a 15% sodium citrate−1.2% KBr-10% citric acid (15% Na-citrate) electrolyte. ZrC, VC and NbC can be isolated by 7% HCl-3% FeCl_3_-ethelyne glycol, 15% sodium citrate-1.2% KBr-30% citric acid electrolyte. Except for VC, all the other carbides, including carbides such as (Cr,Fe)_23_C_6_ and (Cr,Fe)_7_C_3_, could be extracted using the 10% acetyl acetone–1% tetra methyl ammonium chloride–methanol (10% AA) electrolyte.

### 3.2. Maturation Years (1980s–Present)

The Joint Research Committee in Japan [79] discussed the most suitable electrolytes to isolate sulphide inclusions from steels. The 10% AA (10% acetylacetone; 1% tetramethyl ammonium chloride; methanol) electrolyte along with the 4% MS (4% methyl salicylate- 1% salicylic acid- 1% tetramethylammonium chloride- methanol) were found to be the best reagents for this purpose. They successfully demonstrated the extraction of sulphides from Fe-S binary alloy, Fe-Mn-S, Fe-Mn-X-S (where X = C, Si, Al, Ti, Zr, CA, rare earth, O) alloys, SnMn 420 H, Cr-Mo SCM 40 alloy steel and 304 stainless steel. The C content in these steels were in the range of 0.0004–0.22 wt.%. Based on their recommendations, Hinotani et al. [80] reported the isolation of MnS inclusions from Ti-alloyed ultra-low carbon steels (C: 0.0024–0.003 wt.%) using 4% MS electrolysis treatment. 

MnS and AlN inclusions in Fe-3% Si electrical steels could be extracted using two electrolytes composed of 20% NaCl,6% trisodium acetate, 2% citric acid, 5% sodium citrate, 1.2% KBr, 0.5% ascorbic acid and water [81]. It was found than a greater quantity of MnS could be extracted when compared to chemical extraction as described in the same paper.

The electrolytes 10% AA and 2% TEA (2% triethanolamine- 1% tetramethylammonium chloride-methanol) were used to extract oxide particles following the deoxidation of a Fe-10% Ni alloy [82]. It was found that 10% AA could be used to extract particles after Zr, Al and Si/Mn deoxidations, and 2% TEA was used to extract after Mg and Ca deoxidations. The currents used in this work were 150 mV and 45–55 mA with a charge of 1200 coulomb.

**Figure 4:**
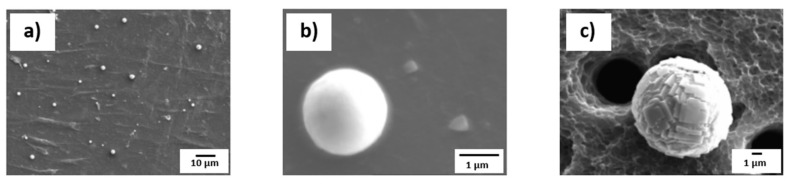
SEM image of the surface filter and the inclusions after extraction of a Fe-20 wt.% Cr alloy using 10% AA, where (**a**,**b**) are particles obtained under a charge of 300 coulomb and (**c**) was a particle obtained under a charge of 600 coulomb [66]. The length scales have been updated in the current paper. Copyright © 2009 Verlag Stahleisen GmbH, Düsseldorf.

**Figure 5:**
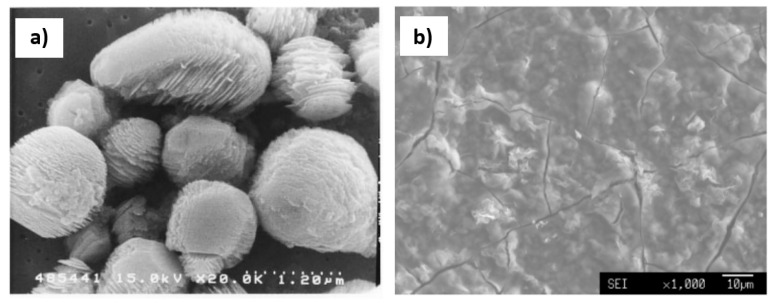
(**a**). Ce-oxide particles extracted by 10% AA at 700 C. It can be seen that the surface of the particles is dissolved. (**b**) Surface of film filter with particles after extraction by 2% TEA at 500 C. It can be seen that particles are almost undetectable due to the precipitated Cr-rich compound covering them [66]. Copyright © 2009 Verlag Stahleisen GmbH, Düsseldorf.

Janis et al. [66] compared the use of 10% and 2% TEA as electrolytes to extract inclusions in Ti and Ce deoxidized high-chromium steels (Fe- 20 wt.% Cr). It was shown that a low charge (300 coulomb) and short extraction times using the 10% AA was the best method to extract complex oxides such as Ce, Ti and Cr-oxides, as seen in Figure 4. They reported that a higher charge resulted in the erosion of the surface of the inclusions. Ti and Cr-based nitrides and oxide-nitrides were also shown to precipitate on these oxides. The degree of insoluble Cr in the residue after extraction was much higher when 2% TEA electrolyte was used when compared to 10% AA. This was due to the precipitation of organic Cr-rich precipitates when under electrolysis using 2% TEA, as seen in Figure 5. When it came to the accurate measurement of soluble and insoluble Ce contents in the metal sample, 2% TEA electrolyte was found to be superior to 10% AA, as it did not dissolve the Ce-oxides as much 10% AA.

Inclusions from high chromium H13 tool steels (0.39–0.42 wt.% C, 4.8–5.3 wt.% Cr) were also extracted using 10% AA electrolyte [83]. A low electric charge of 300 to 500 coulomb was selected for the same reason as that described in the work of Janis et al. [66]. Although the extraction process was successful, the inclusions were found to be covered with Cr, Fe, V and Mo precipitates. This was theorized to have come from the highly alloyed steel during the electrolysis process and the consequence was that the exact inclusion size distribution could not be reliably obtained.

Kanbe [84] identified sulphides of different morphologies such as globular, rod-like and dendritic sulphides from molten 17CrMo4 (0.18 wt.% C, 1.25 wt.% Cr, 0.24 wt.% Mo) steel and elongated sulphides from rolled 17CrMo4 steel using 10% AA non-aqueous solution. All of the experiments were performed by using the following parameters: voltage–150 mV, electric current–40 to 50 mA and electric charge–500 coulomb using 10% AA electrolyte. 

Micro-alloyed precipitates could also be extracted from Grade 100 steel (0.08 wt.% C, 1.8 wt.% Mn) using 10% AA electrolyte [62]. Although the electrolytic extraction proved to be a slower process when compared to the chemical extraction used in the same work, it was reported that the electrolytically extracted particles did not show the presence of any SiO_2_ residue.

Non-metallic inclusions such as MgO and MgAl_2_O_4_ were extracted using acid, halogen-methanol and nonaqueous electrolytes and their stability was compared [65]. The specimens used for the study were Fe- 10 wt.% Ni specimens alloyed with Mg. It was shown that 2% TEA with the addition of Ba was best suited for the extraction of the above-mentioned particles when conducted at 150 mV with a current of 22–42 mA/cm^2^. When compared to 4% MS and 10% AA, 2% TEA with Ba also extracted a higher amount of Mg content from the inclusions. This was theorized to be due to MgO being stable above pH 10. The 2% TEA, 2% TEA-BaO and 2% TEA-Ba had the pH values of 10, 11 and 12, respectively. The pH values for 4% MS and 10% AA were 3 and 5, respectively. The Ba and BaO additions suppressed the dissolution of MgO based on a common ion effect and the dehydration of the 2% TEA. It was also found that the insoluble Mg contents were stable between in the anodic potential range from −201 to −48 mV.

The extraction of inclusions from high manganese high aluminium-alloyed austenitic steels was achieved by using 10% AA electrolyte [85]. The samples in this study were Fe (10, 20 wt.%)-Mn (1,3 and 6 wt.%)-Al alloys (x = 10 and 20 wt.%, y = 1, 3 and 6 wt.%). The current settings were 500 mA for 2 to 3 h (4000 to 5000 coulomb). The extracted inclusions were identified as Al_2_O_3_, AlN, Al(O)N, MnAl_2_O_4_, Al_2_O_3_-(Al(O)N) agglomerates, single Mn(S,Se) particles and Mn(S,Se) core with Al_2_O_3_(-Al(O)N) aggregates. The Se derived from contaminations in the electrolytic metal Mn which was used to prepare the alloys for the study.

Inclusions and clusters in high silicon–non calcium treated stainless steels (Fe–23 wt.% Cr–3 wt.% Si–0.009 wt.% Al and Fe–19 wt.% Cr–12 wt.% Ni–2 wt.% Si–0.003 wt.% Al) were investigated by Bi et al. [86] using 10% AA and 2% TEA electrolytes. The current density used was 35–45 mA/cm^2^. Spinel inclusions (MgO-Al_2_O_3_) were a big constituent in these steels, with MgO occurring in higher concentrations ( >=80 wt.%). As reported earlier [65], pure MgO tends to dissolve in 10% AA electrolyte, but in this reported work, the MgO contents obtained from both 10% AA and 2% TEA electrolytes were reported to agree satisfactorily. This was explained by short electrolytic extraction times of 2.5 to 3 h in the work of Bi et al. Also, similar to the findings in Janis et al. [66], Bi et al. [86] reported that Cr-rich compounds precipitated during the electrolytic extraction of stainless steels using 2% TEA. The precipitates covered the film filter and rendered the observation of inclusions smaller than 2 µm difficult. Therefore, 10% AA electrolyte was preferred for extraction in this work.

Inoue et al. [87] studied the applicability of different non-aqueous electrolytes for the extraction and stability of fine inclusions (<1 µm) such as ZrO_2_, Ti_2_O_3_, TiAl_2_O_5_, Ce_2_O_3_ and CeS. It was found that ZrO_2_, Ti_2_O_3_ and TiAl_2_O_5_ could be extracted, and 4% MS and 10% AA electrolytes were the most suitable non-aqueous electrolytes. It was noted that earlier studies showed that 4% MS was a suitable electrolyte to extract rare earth oxides [79]. However, it was shown that quantity of dissolved Ce_2_O_3_ and CeS inclusions in 2% TEA–Ba was lesser when compared to the use of 4% MS electrolyte. Therefore, it was concluded that Ce_2_O_3_ and CeS inclusions were best suited to extraction using 2%TEA electrolyte dehydrated with Ba like the one used in the previously described study by Inoue et al. (Inoue et al., 2011). Although aqueous electrolytes such as citric acid electrolytes (30% citric acid–15% sodium citrate–1.2% KBr aqueous solution) can be used for the extraction of Zr-based inclusions from steel [88], it was pointed out by Inoue et al. [87] that the Zr ions are prone to hydrolysis. The usage of aqueous electrolytes was, therefore, not advised.

Inclusions from ferroalloys such as FeTi, FeNb, FeSi and SiMn were extracted by using 10% AA electrolyte [89]. It was stated that unlike non-aqueous electrolytes, chemical acid extraction methods can lead to the dissolution of the inclusions. Electrolytic extraction is recommended for the isolation of rare earth metal (REM) oxides which are found in FeTi, Fe, Si and SiMn. The current settings in this study were set between 30 to 40 mA/cm^2^. Inclusions such as Al_2_O_3_ and Ti-Nb-S-O were observed in FeNb. Silicon oxide inclusions were isolated from SiMn. Inclusions of Ferrochromium alloys [90] were also investigated using similar settings to those mentioned in the previous work. The main type of inclusions in low carbon FeCr (0.057 wt.% C, 70.6 wt.% Cr) were Si-Cr-O oxides, and Cr-Mn-S and Ca-O-P inclusions were found to be the main inclusions in high carbon FeCr alloy (8.2 wt.% C and 69.4 wt.% Cr). An accurate 3D investigation of the inclusions in these alloys would help in the optimisation of inclusion characteristics in order to achieve the desired material properties. The inclusion cluster sizes for single inclusions were found to be 2–16 µm and 2 to 77 µm for clusters.

Non-aqueous HCl-based electrolyte with tartaric acid (10% concentrated HCl and 1% tartaric acid in methanol) with a 150 mV vs a standard calomel electrode was used to extract inclusions from industrial 18 Cr stainless steel [91]. Ti and Fe-containing inclusions could be detected. The nucleus was likely titanium oxide, surrounded by Ti and Nb rich nitrides. The extraction of inclusions was successful, but it was advised that a different electrolyte should be selected if steel specimens contain sulphur. This is because sulphides are soluble in acid-based electrolytes.

Inclusions in Mg and Ca-treated high carbon chromium bearing steels (0.99 wt.% C, 1.45 wt.% Cr, 0.3 wt.% Mn) were investigated [92]. As described earlier [65], 2% TEA (150 mV, 40–60 mA, time: 8–12 h) was selected to extract MgO-containing particles from the metal. The identified inclusions were Al_2_O_3_, MgO-Al_2_O_3_, CaO, MgO and CaO-MgO-Al_2_O_3_ system inclusions. 

The electrolytic extraction of rare earth oxides and niobium oxides from 1030 steel (0.309 wt.% C, 0.184 wt.% Si, 0.671 wt.% Mn) was studied [93]. Samples were subject to electrolysis in 10% AA and 2% TEA solutions. The parameters were 3.35 V and 40–45 mA with a charge of 650–800 coulomb. The weights of samples were measured at times of 2, 4, 6, 8, 10, 12, 14 and 16 h. The stability of CeO_2_, La_2_O_3_, Al_2_O_3_, NbO and NbO_2_ powders in 2% TEA was also examined. It was observed that the rate of dissolution was higher in 10% AA when compared to 2% TEA electrolyte. The variation of the dissolution rate for 10% AA as stated in the work of Janis et al. [63] was about 0.0004 to 0.001 g/min. This was much higher when compared to the findings of Bommareddy et al. [93], where the variation was found to be between 0.00094–0.00096 g/min. This was reported to be due to the lower amount of variation in the electrolysis currents used. It was also stated that the slower dissolution rate of the sample in the 2% TEA when compared to the 10% AA would allow for better control with respect to the dissolved sample amount. This would allow for the better assessment of the number of inclusions per volume during the extraction process. It was also found that the 2% TEA left behind lesser residue after the filtration of the dissolved sample. The 10% AA solution left behind a red residue near the sample and in the solution. The removal of the residue was necessary for generating an accurate image of the inclusions and accessing the inclusions present in the sample. From the stability tests in the 2% TEA, it was found that except for a slight loss of NbO and Al_2_O_3_, all the other oxides showed much better stability. Additional EDS results showed that there was no chemical reaction between the inclusions or any formation of complex compounds in 2% TEA. Therefore, it was concluded that 2% TEA was a suitable electrolyte due to the advantages of obtaining a cleaner solute, a slow dissolution and the relatively good stability of all the inclusion oxides.

**Figure 6:**
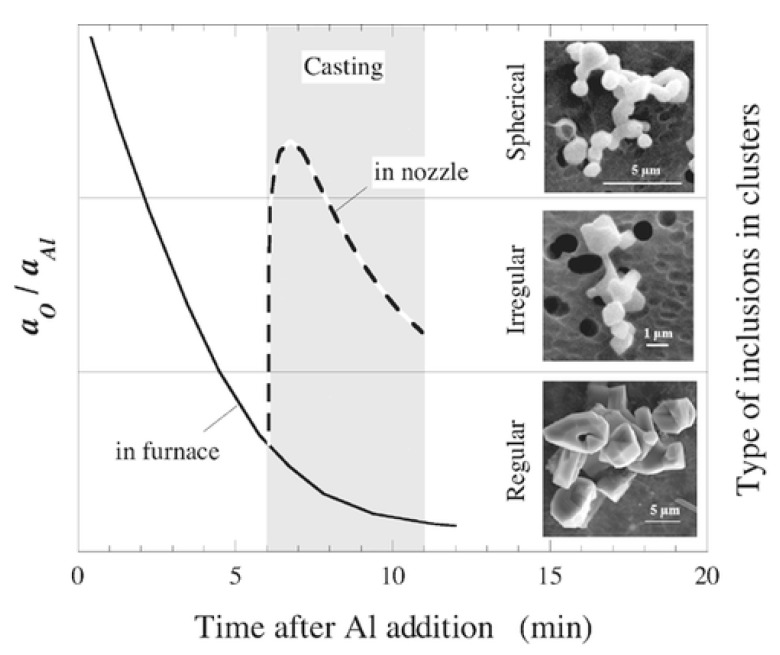
Illustration and SEM micrographs of possible transformation of inclusion morphology in clusters in the melt in the furnace and nozzle during casting as a function of holding time after the addition of Al [19]. © 2015 WILEY-VCH Verlag GmbH & Co. KGaA, Weinheim.

Clusters of Al_2_O_3_ inclusions in liquid (18/8) stainless steel (0.078 wt.% C, 17.9 wt.% Cr) samples can be extracted using 10% AA solution [19]. The current settings used for the study in question were a 700–1000 coulomb electric charge, a 40–60 mA current and a 2–3 V voltage. As seen in Figure 6, SEM micrographs of extracted cluster inclusions help to track how the clustered inclusions formed in the furnace melt changed their shape from spherical to irregular and regular depending on the size and shape of alumina inclusions, which themselves depend on the ratio between the local activities of oxygen (a_O_) and aluminium (a_Al_).

**Figure 7:**
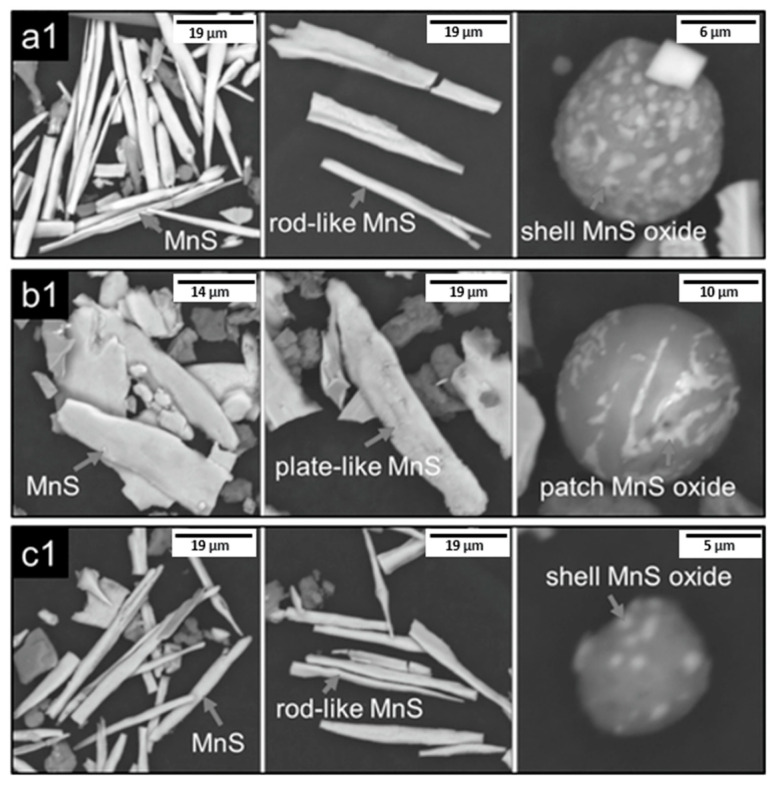
3D morphology of typical MnS inclusions in rail extracted using non-aqueous electrolyte; (**a1**,**b1**,**c1**) represent the rail head, rail waist and rail bottom, respectively [18]. The length scales have been updated in the current paper. Copyright 2016 WILEY-VCH Verlag GmbH & Co. KGaA, Weinheim.

Zhang et al. [18] extracted and compared MnS inclusions in continuous bloom and rolled heavy rail steels (0.77 wt.% C, 0.63 wt.% Si, 0.87 wt.% Mn, 0.028 wt.% Cr), as seen in Figure 7. The electrolyte selected was non-aqueous 1% 4-methyl ammonium chloride, 5% triethanolamine, 5% glycerin and 89% methyl alcohol at a current ≤ 100 mA/cm^2^, as proposed in the work of Keming and Ruiming [94]. The electrolysis was performed under an Ar atmosphere to prevent the oxidation of the sample. This allowed for extraction of both acid–resistant inclusions, such as oxides and nitrides, and acid non-resistant inclusions such as MnS.

**Figure 8:**
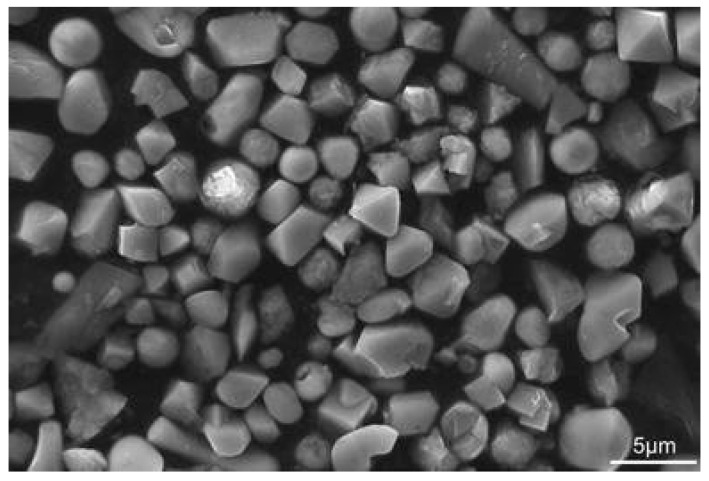
Morphologies of typical sulphide and nitride inclusions in low-sulphur spring steels [95]. Copyright 2016, Springer Nature.

An improved methodology used to filter out inclusions after electrolytic extraction was reported by Liu et al. [95]. Here, MnS, oxide and TiN complex inclusions, as seen in Figure 8, were observed in low-sulphur spring steels (0.55 wt.% C, 1.5 wt.% Si, 0.67 wt.% Mn, 0.7 wt.% Cr) which were deoxidised by Si-Mn. The electrolyte used was 1% 4-methyl ammonium chloride, 5% triethanolamine, 5% glycerin and 89% methyl alcohol. The current density was kept between 40–60 mA/cm^2^. The temperature of the electrolyte was kept between 268 K to 278 K for 8 h. Conventionally, the liquid containing the inclusions is filtered, and, as a result, most of the inclusions are dispersed and the impurities in the liquid gives a black colour to the solution. It was proposed that the inclusions were collected and elutriated several times with alcohol in a water glass until the inclusions were gathered and appeared. The water glass was then covered and subject to drying at room temperature for 8 h. The inclusions were then transferred directly to a double-sided carbon tape. It was shown that this method resulted in the collection of more inclusions, especially of the MnS type, that collection was easy to carry out and that the observations were improved regarding the morphology, size and characterisation of the inclusions.

**Figure 9:**
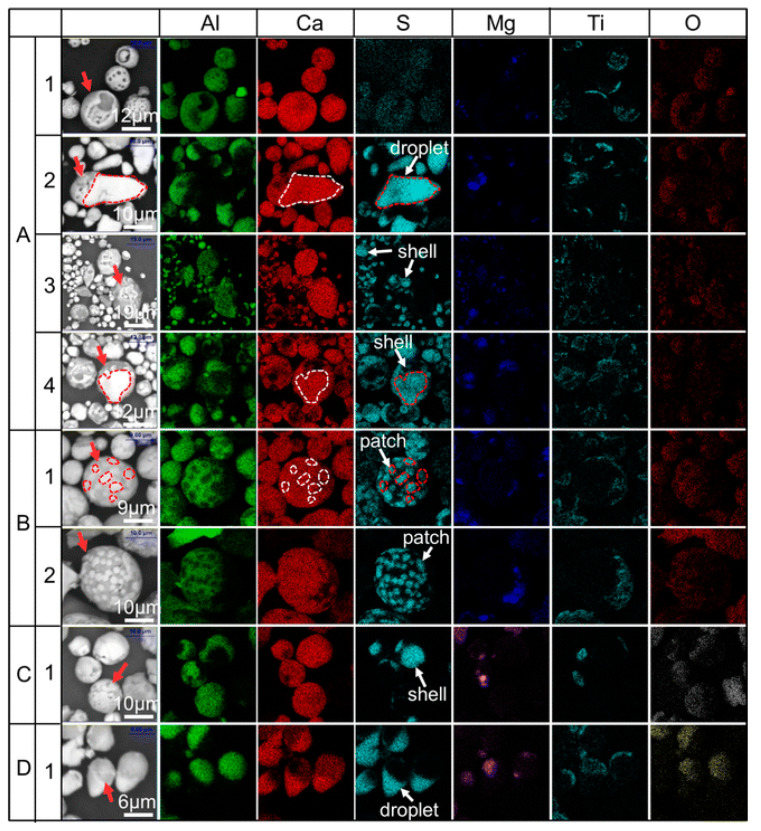
SEM images which show the 3D morphology of inclusions and the corresponding mapping of chemical elements in linepipe steel [96]. Copyright 2017, Springer Nature.

Ca–CaS type inclusions were extracted using precipitates from line pipe steels (0.046 to 0.061 wt.% C, 1.88 to 2.08 wt.% Mn steel) [96], as seen in Figure 9. The electrolyte chosen was again the 1% 4-methyl ammonium chloride, 5% triethanolamine, 5% glycerin and 89% methyl alcohol at a current of 100 mA/cm^2^. Here, the electrolysis was performed under a nitrogen atmosphere to prevent the oxidation of steel. This study showed that 3D characterisation after electrolytic extraction enabled the proposal of a new kind of calcium treatment. Also, it allowed for the classification of different morphologies of CaO–Al_2_O_3_ system inclusions and CaS inclusions.

**Figure 10:**
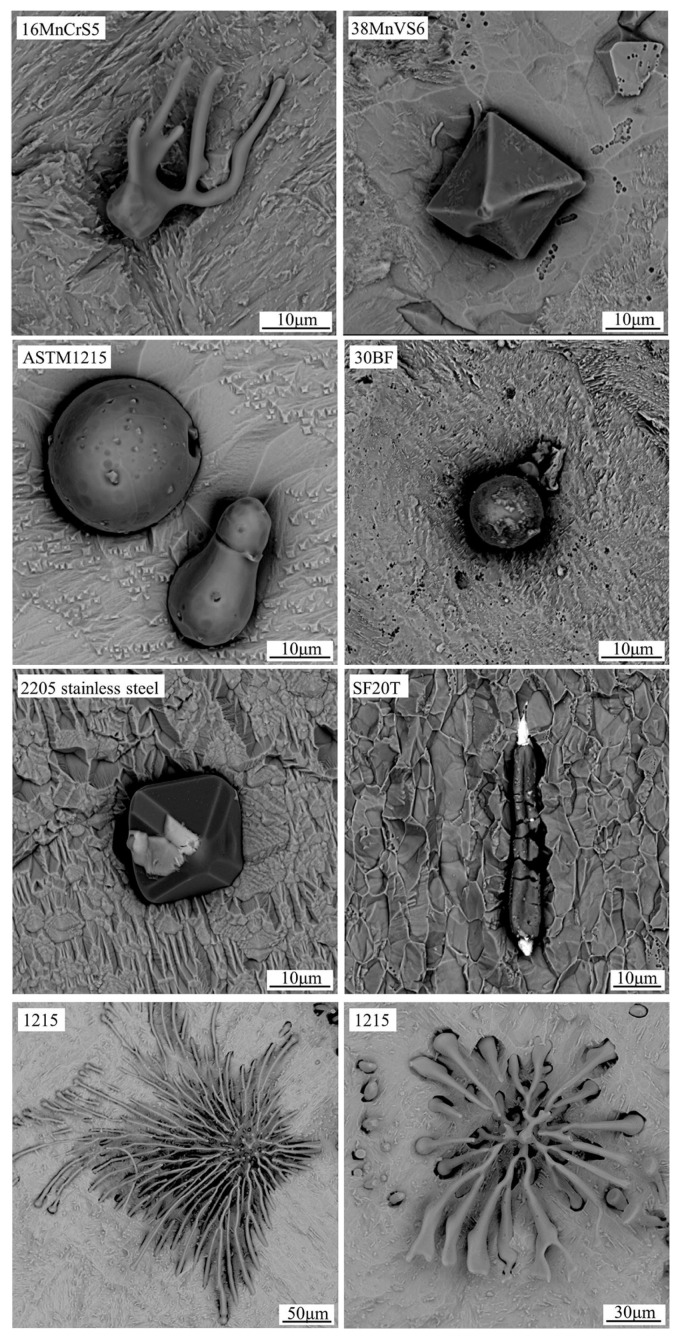
Morphologies of sulphides in different steel grades, as seen in Zhang et al. [97]. Copyright 2019, Springer Nature.

A different route from the methodologies described above involves partial extraction after electrolytic etching, as performed by Zhang et al. [97], who extracted sulphide inclusions from sulphur-bearing steels (16MnCrS5, 0.02 wt.% S). The influence of current density, time and temperature on exposure degree of sulphide inclusions were also explored. The electrolyte used was 10% AA in conditions of 37.5–52.5 mA/cm^2^ for 30–35 min. The extraction was performed at various temperatures, and it was found that increasing the temperature in turn increases the surface activity of sulphides. This leads to an increase in electrolytic conductivity and therefore a loss of sulphides. Therefore, the etching experiments were conducted at −10 °C to 0 °C so as to maintain the morphology of the inclusions. Figure 10 shows the morphology of sulphides found in different steel grades, as reported in their work.

Ti based inclusions and particles were extracted from Ni-based steel alloy 825 (0.76 to 0.8 wt.% C, 38 wt.% Ni, 20 wt.% Cr) for 3D characterization, especially TiN particles and clusters [98,99]. The electrolyte used was 10% AA under conditions of 4 V, 40–60 mA and a charge of 1000 coulomb. The electrolyte worked well by not dissolving the TiN and allowing for the observation of clusters in the size range of about 5.6 to 11 μm. The same electrolyte was used at a charge of 500 coulomb to extract Al_2_O_3_ and Al_2_O_3_-based inclusions from the same alloy system [99]. 

Wang et al. [100] showed that 10% AA (60–70 mA, 4.2–5 V, 500 coulomb) can successfully be used to extract and study inclusions in ferroalloys. It was found that the most harmful inclusions were Al_2_O_3_ and high Al_2_O_3_ inclusions in FeV alloys, SiO_2_ and high SiO2-containing inclusions in FeMo, Al_2_O_3_ and SiO_2_ inclusions in FeB alloys and MnO_3_–Cr_2_O_3_, Al_2_O_3_ and Cr_2_O_3_-based inclusions in FeCr alloys. 

Du et al. [101] compared the used of 10% AA and 2% TEA electrolytes (3.2–4.3 V, 50–70 mA, 500–1000 coulomb) to extract inclusions from 316 L steel stainless steel which had been modified with the addition of CaSi. It was found that there were no differences in the compositions of the oxide inclusions extracted by both electrolytes, with their morphology and size also being similar. The inclusions that could be identified in 316 L were elongated MnS, MnS sulphides with hard oxide cores, undeformed irregular oxides and elongated sulphides with hard oxide cores. 

Nabeel et al. [102] investigated inclusions in medium Mn AHSS. The electrolyte used for the extraction process was 10% AA (50 to 60 mA, 2.7 to 3.5 V). It was shown that increasing Mn resulted in a higher number of inclusions. The main inclusions in medium Mn AHSS were Al_2_O_3_, MnS, AlN and AlSiMn-oxide. It was shown that larger numbers of inclusions could be detected by 2D automated SEM methods when compared to 3D extractions. This was theorized to be because a greater number of smaller-sized inclusions was observed during the 2D analysis. Therefore, it was suggested that a better representation of inclusions can be determined by automated 2D methods, particularly if there is an inhomogeneous distribution of inclusions in the samples. The electrolytic extraction methods detected higher MnS and decreased AlN inclusions with increased Mn content in steel. This was the opposite to what was observed using the 2D automated method. This could be due to the difference in the analysed size ranges. Another reason could be that in the electrolytic extraction method, all inclusions can be classified as four classes, irrespective of if they are single or multiphase. The multiphase inclusions is added to the class that represents their dominant phase. It is not possible to detect the core of inclusion if a multicomponent inclusion is wrapped by another. But in 2D analysis, cross-sections are exposed; e.g., the Al_2_O_3_–MnS inclusions had an Al_2_O_3_ core and MnS outer layer. This result would be classified as MnS using the EE method but as Al_2_O_3_ using the automated method. However, the detection of the shapes of inclusions was superior when using 3D extraction techniques. 

**Figure 11:**
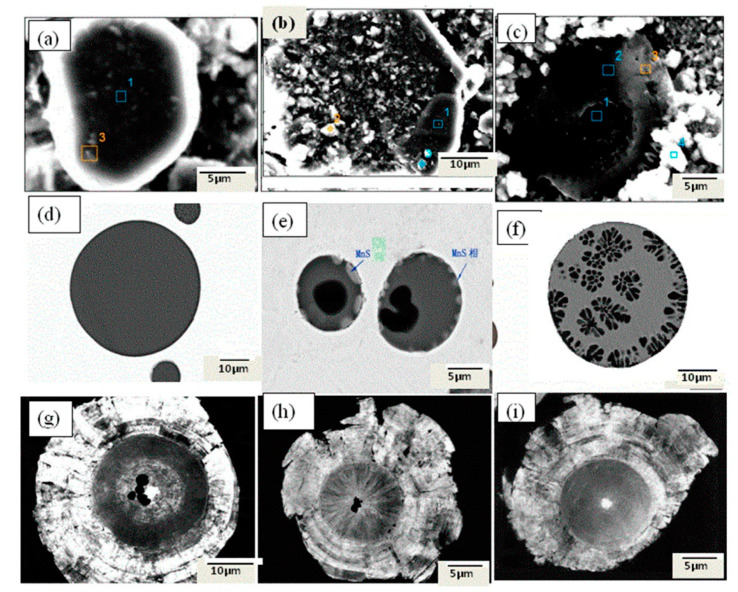
Exposed inner structures of extracted inclusions as cut using the RTO technique: (**a**) single Al_2_O_3_-based inclusion, (**b**) aggregated Al_2_O_3_-based cluster, (**c**) CaO-SiO_2_ based inclusion, (**d**) homogeneous spheroidal SiO_2_-MnO-CaO-Al_2_O_3_ inclusion, (**e**) SiO_2_-rich phases precipitating within the spheroidal SiO_2_-MnO-CaO- Al_2_O_3_ inclusion and a spindle-like MnS phase on the inclusion surface, (**f**) tiny dendritic structure SiO_2_-rich phases precipitating both on the SiO_2_-MnO-CaO- Al_2_O_3_ inclusion surface and as inner inclusions, (**g**–**i**) one or several rare-earth oxides acting as a crystal core in the spheroidal graphite [104].

One way of exposing the cores of inclusions is by using the room temperature organic (RTO) method demonstrated in the work of Guo et al. [103]. They used electrolytic methods to investigate non-metallic inclusions in ultra-low carbon IF steel. The electrolyte used for the study was a solution of 4–10% glycerine, 4–10% triethanolamine and 0.1–5 wt.% tetramethylammonium chloride. The electrolysis was carried out at 0.5 °C and 200 mA/mm^2^ for 4 h. After the electrolytic extraction, the extracted inclusions were laid on a clean copper plate in a monolayer. This was then used as a cathode, with another pure copper plate used as an anode. An electrolytic solution with a similar composition to that mentioned above was used at room temperature. The procedure is such that a current lower than 500 mA is supplied using a direct current power source. Cu then gets deposited on the inclusions in a layer-by-layer manner from the anode. The inclusions on the cathode copper plate are finally completely wrapped by the deposited Cu. The cathode copper plate is then ground using a very fine grit paper (>1000 grit). The result is that the inclusions which were wrapped in the copper can be cut and their inner structures are exposed. The RTO technique has also been used previously by Guo et al. [104] to determine the interior structures of Al-killed, Si-killed and ductile cast iron steels, as seen in Figure 11.

Guo et al. [104] mentions that the inner structure on an inclusion must be studied so as to determine the formation and growth mechanisms of inclusions. Traditional 2-D metallographic methods could be used to obtain information regarding the inner structures of inclusions, but they come with the problems of high contingency and low efficiency. It was for this reason that Guo et al. developed the room temperature organic (RTO) technique to wrap and cut the collected inclusions for the convenient and effective investigation of their inner structures. It was found that, other than in the case of Cu, the matrix is not measured around the inclusions when using the RTO technique. The RTO method allowed for a more accurate analysis when compared to the traditional 2-D method.

Luo et al. [105] investigated the number and size distribution as well as the morphology of nitride precipitation during the manufacturing of non-oriented silicon steel (2.8–3.1 wt.% Si, 20–26 ppm of C) and also took account of different cooling rates during solidification under lab conditions. The electrolyte used was 5% TEA at 100 mA/cm^2^. The electrolyte was kept refrigerated below 263 K. Nitride types such as pure nitrides, oxynitrides and nitrides with oxysulphides could be investigated.

Luo et al. [106] investigated the inclusions in oriented silicon steels (3.1 wt.% Si, 0.031 wt.% C); 5% TEA at 100 mA/cm^2^ was also used for these steels. The inclusions detected were alumina and alumina silicate oxides with spherical, elliptical and irregular shapes and compounds of AlN and sulphides such as Cu-S, TiS and MnS.

**Figure 12:**
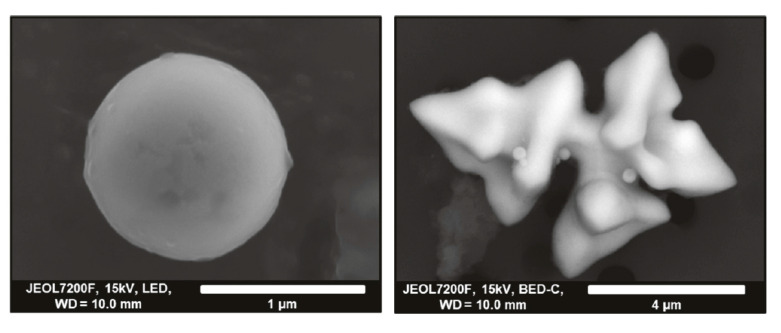
Electrolytically extracted inclusions using the choline chloride solution as mentioned in Mayerhofer et al. [11]. Adapted from Ph.D thesis of Alexander Mayerhofer [11].

Mayerhofer et al. [68] demonstrated the electrolytic extraction of inclusions on a Fe-Mn-O master alloy steel. An ionic liquid (choline chloride / urea = approx. 1:1) with dimethylformamide additive was used at 60 °C, 0.1 A and 15 V for 180 min. It was shown that the non-aqueous electrolyte showed no negative effect on (Fe, Mn) oxides in terms of morphology and chemistry, as seen in Figure 12.

Recently, Guo et al. [107] demonstrated the use of 10% AA at 40–60 mA, 3.5 V and a charge of 500 coulomb for the extraction of sulphide inclusions from tool steels and stainless steels (tool steels: 42CrMo4 and 13HMG (0.42 wt.% C and 0.15 wt.% C, respectively) and stainless steels: 3R65 and 316L (0.03 wt.% C and 0.02 wt.% C, respectively)). The steel samples were heat treated at about 900 °C to dissolve any carbides which might be in the steel. They were subsequently rapidly cooled to avoid the formation of carbide on inclusions. It was observed that the inclusions that were extracted from tool steels with an appreciable amount of carbon (>0.15 wt.% C) and were covered with carbides. However, Guo et al. also observed that additional heat treatment prevented carbide formation. It was found that 900 °C heat treatment for 5 min dissolved any carbides on MnS, thereby allowing for a precise investigation. The heat treatment was shown to not affect the morphology and fragility of sulphides. Further, it was also observed that the preparation of the samples, electrolytic extraction process or the thickness of the metal layer that was dissolved did not have any noticeable effects on the morphology of sulphides in deformed steels.

Sidorova et al. [108] used a fast electrolytic extraction method using 10% AA electrolyte so as to not cause the dissolution of inclusions found in low carbon pipeline steels (0.05% C). The electric parameters used were 40–60 mA at voltages of 2.9–3.8 V. Complex inclusions, such as pure CaS inclusions or oxide cores completely covered by a CaS layer, CaO-Al_2_O_3_-MgO inclusions partially covered by a CaS layer, oxide inclusions containing small precipitations of CaS and TiN inclusions and oxide inclusions completely covered by TiN or pure TiN inclusions could be extracted. The work also demonstrated the evaluation of the initial stages of local corrosions that caused the destruction of the metal matrix surrounding the inclusions on the surface of the steel samples using the same electrolyte without the application of an electric current.

## 4. Comparison of Different Extraction Approaches

A summary of the extraction methods, inclusion types and steel grades discussed in the earlier sections is presented in Table 1. Only the methods used after the 1980s were documented as it was only after this period that the techniques matured. The methods presented are more or less used in the same configurations in the present period. It is clearly seen from the table that chemical extraction methods have mainly been used for low carbon and low alloyed steels. Electrolytic extraction techniques have proved to be the most versatile by covering all of the different grades of steel. The use of chemical methods has mainly been limited to the extraction of stable oxides. Although halogen-based extraction is more versatile in terms of the range of extracted inclusions, it requires further safety measures in laboratory settings. Modified chemical extraction methods, such as the sequential method using Nital, as described in this paper, have aided in the extraction of less stable inclusions such as sulphides and have therefore extending their use. Electrolytic extraction techniques have been the preferred techniques for isolating a range of inclusions from higher alloyed steels. The most commonly used reagent for the extraction of various types of inclusions over a wide range of steel grades has been 10% AA electrolyte. Additionally, 2% TEA has been used as an alternative to 10% AA, as it leaves behind less residue after the filtration process, as reported by Bommareddy et al. [93]. However, it comes with drawbacks, such as the additional precipitation, for example, organic Cr rich precipitates, produced during the electrolysis process, as reported by Janis et al. [66].

## 5. Conclusions

In summary, extraction methods for the isolation of inclusions have a long history that extends for over a century. The earliest methodologies required the use of complicated apparatus for conducting extraction methods. Acid extraction methods required relatively simpler set-ups, while halogen methods and electrolytic extraction methods required more complicated set-ups. Acid extraction techniques were found to be good for extracting stable inclusions such as alumina but came with the disadvantage of the dissolving of inclusions such as sulphides. In this regard, halogen techniques and electrolytic extraction techniques were found to be superior for extracting a wide range of inclusion types. The main disadvantage of using halogen techniques was the handling of hazardous chemicals in the laboratory environment. Therefore, with the development of better equipment, electrolytic extraction methods became a popular method for the extraction of inclusions from various grades of steel. The usage of non-aqueous electrolytes has been favoured, as they do not undergo hydrolysis, which could, in turn, attack chemically sensitive inclusions, as in the case of aqueous electrolytes. Extraction techniques matured after 1980s and the significant methods are documented in Table 1. The current settings used for electrolysis also have a significant influence on the extraction process. It was generally observed that the charge required to successfully isolate inclusions must be proportional to the amount of alloying content in the steels. The most widely used reagent for electrolytic extraction in the reviewed articles was 10% AA. By controlling the current parameters, this reagent could be used for extracting inclusions in a wide range of steel compositions. The sequential extraction technique using Nital is promising method because it allows for the recovery of a wide range of inclusions, including sulphides. This method has the advantages of being economical in terms of cost and requiring simple apparatus for conducting the extractions. As of now, sequential extraction has only been used for low carbon alloyed steels and there is scope to explore its usage in higher alloyed steels. 

Considering the growing demands for high steel cleanliness, a detailed characterization of non-metallic inclusions is of the utmost importance. Extraction techniques enable a detailed insight in the morphology of steel samples, providing essential information for the interpretation of particle formations and modifications at a specific stage in the production process. These techniques are not only powerful tools in terms of inclusion analytics but also contribute to a better understanding and interpretation of thermodynamics and kinetics in the field of inclusion metallurgy. In combination with advanced possibilities in SEM/EDS measurements, extraction will likely be able to further extend its significance to the analysis of smaller inclusion sizes.

## Figures and Tables

**Table 1:** Extraction methods after 1980s.

					Inclusions			
Main Content	Steel Types	Method		Reagent	Oxides	Nitrides	Sulphides	References
0–0.01 wt.% C	Pure iron	Chemical		5% nital sequential	●	●	●	Lab
	Electrolytic iron	Chemical		5% nital sequential	●	●	●	Lab
	Plain carbon steel		Electrolytic	10% AA 4% MS			●	[79]
	Ti-stabilised IF-Steel	Chemical		5% nital sequential	●	●	●	Lab
	Ultra-low carbon IF steel		Electrolytic	4–10% TEA	●	●	●	[103]
0.02–0.1 wt.% C	Low carbon aluminium killed steel, low carbon aluminium and silicon killed steel	Chemical		Hot HCl	●			[60]
	Low carbon aluminium killed steel		Electrolytic	4–10% TEA	●	●	●	[104]
	Si killed steel		Electrolytic	4–10% TEA	●	●	●	[104]
	Line-pipe steels		Electrolytic	5% TEA	●		●	[96]
	Low eutectoid steels	Chemical		5% nital sequential	●	●	●	Lab
	Low carbon Oil-pipeline steels		Electrolytic	10%AA	●	●	●	[108]
	Grade 100 micro alloyed steel	Chemical	Electrolytic	C: Hot HCl E: 10% AA	●	●		[62]
0.1–0.2 wt.% C	Medium Mn AHSS		Electrolytic	10% AA	●	●	●	[102]
	Rimmed steel	Chemical		Hot nitric acid	●			[40,41]
	Medium carbon aluminium killed steel	Chemical		Hot HCl	●			[60]
	Alloyed steel	Chemical		5% nital sequential	●	●	●	[67]
	13HMF		Electrolytic	10% AA	●	●	●	[107]
	1015	Chemical		HCl	●			[61]
	Mid-C-steel	Chemical		5% nital sequential	●	●	●	lab
	Low eutectoid steel	Chemical		5% nital sequential	●	●	●	lab
0.2–1 wt.% C	Alloyed steel	Chemical		5% nital sequential	●	●	●	[11,67]
	1030		Electrolytic	10% AA 2% TEA	●			[93]
	SiMn killed spring steels		Electrolytic	5% TEA	●	●	●	[95]
	SiMn killed heavy rail steels		Electrolytic	5% TEA	●	●	●	[18]
>1% C	Ductile cast iron		Electrolytic	4–10% TEA	●	●	●	[104]
	Electrical steels		Electrolytic	20% NaCl, 6% trisodium acetate, 2% citric acid; sodium citrate 5%, KBr 1.2%, 0.5% ascorbic acid, water		●	●	[81]
	Oriented silicon steel		Electrolytic	5% TEA	●	●	●	[106]
	Non-oriented electrical steel		Electrolytic	5% TEA	●	●	●	[105]
Lab alloys	Fe-Mn-O		Electrolytic	Ionic liquid (choline chloride / urea = approx. 1:1)	●	●	●	[68]
	Fe-10 wt% Ni		Electrolytic	10% AA 2% TEA	●			[82]
	Fe-10 wt% Ni		Electrolytic	2% TEA 2% TEA-Ba	●			[65]
	Fe-20 wt% Cr		Electrolytic	10% AA 2% TEA	●			[66]
	Fe- 10 to 20% Mn–1–3% Al TRIP alloy		Electrolytic	10% AA	●		●	[85]
1–5 wt.% Cr Steels	SCM440 medium carbon chromium molybdenum alloy		Electrolytic	10% AA 4% MS			●	[79]
	17CrMo4		Electrolytic	10% AA			●	[84]
	H13 tool steel		Electrolytic	10% AA	●		●	[83]
	High chromium bearing steels		Electrolytic	2% TEA	●			[92]
	42CrMo4		Electrolytic	10% AA	●		●	[107]
>5 wt.% Cr, > 10 wt.% Ni Steels	SUS404		Electrolytic	10% AA 4% MS			●	[79]
	316L		Electrolytic	10% AA	●			[86]
	316L		Electrolytic	10% AA 2% TEA	●			[101]
	316L		Electrolytic	10% AA	●		●	[107]
	Industrial stainless steel		Electrolytic	Non-aqueous HCl-based electrolyte with tartaric acid	●	●		[91]
	18/8 stainless steel		Electrolytic	10% AA	●			[19]
	Ni-based highly alloyed 825		Electrolytic	10% AA	●	●		[98,99]
	3R65		Electrolytic	10% AA	●		●	[107]
Ferroalloys	FeTi		Electrolytic	10% AA	●			[89]
	FeNb		Electrolytic	10% AA	●		●	
	FeSi		Electrolytic	10% AA	●			
	SiMn		Electrolytic	10% AA	●			
	FeCr		Electrolytic	10% AA	●		●	[90]

## Data Availability

Not applicable.

## Nomenclature

**Symbol**	**Description**
SEM-EDS	Scanning electron microscope–energy dispersive spectroscope
2D	2-dimensions
3D	3-dimensions
ppm	Parts per million
Wt.%	Weight percent
AA	Acetyl acetone-tetra methyl ammonium chloride-methanol
TEA	Triethanolamine-tetramethylammonium chloride-methanol
MS	Methyl salicylate-salicylic acid-tetramethylammonium chloride-methanol
REM	Rare earth metals
